# A Reference Handbook of Medical Sciences

**Published:** 1886-10

**Authors:** 


A Reference Handbook of the Medical Sciences. Embracing the entire range of Scientific and Practical Medicine and Allied Science by various writers. Vol. III., FAC to HYS. 813 pages. Illustrated by six chromo-lithographs and 718 fine wood engravings. Edited by Albert H. Buck, M. D. Supplied to subscribers only. Price per vol., cloth, $6.00; sheep, $7.00; half Morocco, $8.00. William Wood & Company, New York.

Those who have carefully examined the contents of the two preceding volumes of this elaborate work will be prepared to expect a like order of excellence in the contributions to the present volume, and they will not be disappointed. In some respects this collection exceeds in importance what has already been presented to the profession, not only in the nature of the subjects, but in the manner of treatment and the style of illustrating the several topics. The perfection and elegance of the colored plates which accompany these different articles must challenge the admiration of all who appreciate artistic excellence, while the numerous well-executed engravings found throughout the 813 pages of this volume add greatly to the better comprehension of the views of the various authors.

A most acceptable service has been rendered by the exhaustive treatment of the different forms of hernia in the papers of Dr. H. H. Mudd and of Dr. Middleton Michel, which, in connection with the elaborate article on the groin, by Dr. R. L. MacDonnell, cover this ground completely. The very acceptable offering of Dr. N. Senn upon hodrocele fills up the measure of expectation in the surgery of this region most satisfactorily. A thorough investigation of the diseases of the gall-bladder and ducts, by Dr. J. H. Musser, affords a proper basis for the appreciation of operative proceedings, and forms the complement of papers on Cholecystectomy, Cholecystotomy and Duodeno-Cholecystostomy, by Dr. J. McF. Gaston, in volume II. of this work.

In this connection it may be noted that in a paper read at the recent meeting of the British Medical Association, Mr. Willet endorsed the desirability of making an entero-cystic fistula in obliteration of the common bile duct, and states that "in future cases of the kind he would certainly do so. There were two sites at which this fistula might be formed: one was at the junction of the first and second parts of the duodenum; the other was where the ascending joined the transverse colon."*

The suggestion of the former operation, and its demonstration on dogs by Dr. Gaston, supported with similar experiments of Golzi, and the application of the latter in the human subject by Winiwarter, gives an assurance of the practicability of those processes without the inconvenience of an external fistula.

A concise but comprehensive paper on the fallopian tubes, by Dr. W. Gill Wylie, does not leave anything to be said on this subject, even by Lawson Tait.

The differential diagnosis of organic valvular disease of the heart, by Dr. F. Peyre Porcher, brings out the most important points, and the demonstration is reduced to a formula which materially assists the examiner. An article on Fungi by the same author is richly illustrated and is instructive. A paper on Fractures, by Dr. Lewis A. Stimson, presents figures of the apparatus employed generally in the injuries of the thigh bone; and perpetuates an error in the principle of extension by weights and pulleys orby elastic bands and springs. A bone, having sustained

*Medical Record, page 269. September 4, 1886.

such breakage as to admit of the separation of its extremities, requires only that force which shall maintain them in apposition, and this can be effected more satisfactorily by a fixed and limited extension than by a variable force.

A well illustrated paper on Harelip, by Dr. Albert Vanderveer, stands prominently among the practical contributions to surgery. When the frequency of this congenital defect, with the failures in many instances to remedy it, are considered, in connection with the high estimate put upon the restoration of the normal relations and appearance, the profession ought to appreciate the improved process herein delineated for its correction.

Space is not allowed for even a passing reference to the numerous other valuable papers which testify to the fitness of their authors for the work assigned them by the editor.

